# The Effect of Hybridized Carbon Nanotubes, Silica Nanoparticles, and Core-Shell Rubber on Tensile, Fracture Mechanics and Electrical Properties of Epoxy Nanocomposites

**DOI:** 10.3390/nano9071057

**Published:** 2019-07-23

**Authors:** Ankur Bajpai, Stéphane Carlotti

**Affiliations:** LCPO, Bordeaux INP, University of Bordeaux, CNRS, UMR 5629, F-33600 Pessac, France

**Keywords:** Epoxy, nanocomposite, fracture mechanics

## Abstract

The paper investigates the effect of adding a combination of rigid nanoparticles and core-shell rubber nanoparticles on the tensile, fracture mechanics, electrical and thermo-mechanical properties of epoxy resins. SiO_2_ nanoparticles, multi-walled carbon nanotubes (MWCNT’s), as rigid nanofillers, and core-shell rubber (CSR) nanoparticles, as soft nanofillers were used with bisphenol-A-based epoxy resin. Further, the rigid fillers were added systematically with core-shell rubber nanoparticles to investigate the commingled effect of rigid nanofillers and soft CSR nanoparticles. The resulting matrix will be broadly evaluated by standard methods to quantify tensile, fracture mechanics, electrical, and thermal properties. The results show that the electrical conductivity threshold is obtained at 0.075 wt. % for MWCNT-modified systems. For hybrid systems, the maximum increase of fracture toughness (218%) and fracture energy (900%) was obtained for a system containing 5 wt. % of CSR and 10 wt. % of SiO_2_. The analysis of the fracture surfaces revealed the information about existing toughening micro-mechanisms in the nanocomposites.

## 1. Introduction

Epoxy resins belong to a class of highly cross-linked thermoset polymers used most often with reinforcing fibers in a wide range of composite applications, e.g., automotive, aerospace, and pressure vessels [1]. They show a high specific strength, high modulus, dimensional stability, and low creep. At the same time, the high crosslink density makes them intrinsically brittle materials leading to very low fracture energy. The fracture energy can be increased by adding different modifiers to the epoxy resin, e.g., carboxyl-terminated butadiene acrylonitrile (CTBN) [2,3]. Nevertheless, these modifiers are unfavourable to strength and the glass transition temperature (T_g_) [4] of modified systems. A newer type of modifier is core-shell rubber (CSR) nanoparticles. They consist of a rubbery core and an epoxy-compatible shell material, which is only a few nanometers thick. These modifiers-induced the toughening mechanisms of rubber-based modifiers, e.g., cavitation, plastic void growth, and eventually shear yielding [5] together with a better adherence to the matrix due to a modified shell material [6,7]. The addition of rigid fillers like MWCNT’s [8], TiO_2_ [9], Al_2_O_3_ [10], Glass [11], and SiO_2_ [12,13,14] can improve the strength and modulus of epoxy nanocomposites while also increasing the fracture toughness without decreasing the glass transition temperature of the nanocomposites. Unmodified epoxy materials have high resistivity and come into the group of insulator polymeric materials. Various fillers can be incorporated in epoxy to reduce their resistivity and can be used for various applications electrical applications. The inherent conductivity of fillers, their aspect ratio, interactions between filler surface and polymer, their dispersion and alignment are critical parameters to obtain the conductivity and their percolation threshold [15,16]. The CNT based polymer nanocomposite attracted researchers because of their superior mechanical and electrical properties. Previous studies revealed that mixing a slight amount of CNT’s (˂1 wt. %) to epoxy matrix can increase the electrical, mechanical and thermal properties without affecting the process-ability of composites [17]. The presence of CNT’s can cause new mechanisms for energy dissipation during the fracture process, leading to increase in fracture toughness of epoxy resins. Some toughening mechanisms reported by several researchers are mainly pull-out, crack bridging, plastic void growth around bonded CNT’s and interfacial bonding [18,19,20,21]

The objective of this study is to show the effect of SiO_2_, multi-walled carbon nanotubes (MWCNT’s) and core shell rubber (CSR) nanoparticles loading on the mechanical, electrical, and thermal properties of epoxy nanocomposites. The differential scanning calorimetry (DSC) and dynamic mechanical analysis (DMA) was performed to evaluate thermal performances. Tensile tests and fracture test were performed to evaluate mechanical performances. Four-point probe method is used to calculate the electrical conductivity of tailored nanocomposites. Microscopic observations were used to probe the dispersion of CNT’s, SiO_2_ nanoparticles, CSR and obtained nanocomposites toughening mechanisms. The obtained results are to be used as the matrices for the development of innovative low-, mid- and high-pressure tanks based on epoxy/glass fibers composites.

## 2. Materials and Methods 

The EPIKOTE^TM^ Resin L1100 (diglycidyl ether of bisphenol A) liquid epoxy resin and EPIKURE curing agent 943 (amine hardener) produced by Hexion, are used in the present work. The resin and hardener are used in the ratio of 100: 23. The mixed viscosity of the system is 250 mPa.s at 25 °C [22]. The MWCNT’s were supplied by Nanocyl, Belgium in the form of 2 wt. % masterbatch in bisphenol-A based epoxy resin. The CSR nanoparticles used in this work are Kane Ace MX267 supplied by Kaneka Belgium NV which has epoxy equivalent weight of 269 g.eq^−1^. The material supplied is in the form of a master-batch which was a 37 wt. % concentrate of CSR toughening agent in unmodified liquid epoxy resin based on bisphenol-F based epoxy system, the core of the material is polybutadiene based rubber [23]. The SiO_2_ nanoparticles (Nanopox F520) supplied by Evonik Industries in the form of master-batch which contains 40 wt. % of SiO_2_ nanoparticles dispersed in bisphenol-F based epoxy system. The epoxy nanocomposites were cured using a two-step curing cycle: (1) 85 °C for 0.5 h, (2) 120 °C for 2 h. Table 1 shows the different systems prepared in this work.

## 3. Experimental Methods

### 3.1. Differential Scanning Calorimetry

The DSC experiments were performed on a Q100 (TA instruments, New Castle, DE, USA) system to determine the T_g_. Firstly, the cured sample material was weighed (~5–10 mg) and placed in a crucible, sealed with lids with the help of crucible sealing press. In the first cycle, the sample was heated from 25 °C up to 200 °C, cooled down to room temperature, and again heated to 200 °C with a heating rate of 10 °C/min.

### 3.2. Dynamic Mechanical Thermal Analysis

In the present study, the storage modulus, the loss modulus, and tan δ of all the bulk samples were measured by dynamic mechanical thermal analysis using a DMA RSA3 machine from TA Instruments, New Castle, DE, USA in 3-points bending mode operating at 1 Hz, on specimens. The glass transition temperature, T_g_ of the bulk epoxy samples was determined by the peak value of tan δ curve. The temperature range was set from 0 °C to 180 °C with a heating rate of 3 °C/min. 

### 3.3. Electrical Conductivity

For some applications in anti-static environments, an inherent electrical conductivity of the composite material is required to dissipate the electrical discharge. The volume conductivities of the CNT composites were determined via a 4-point measurement according to DIN EN ISO 3915 (1999). The sample size was approximately 4 × 4 × 8 mm^3^. In all cases, the samples were cut from the middle of dumbbell-shaped specimens and the electrical resistances of the composites were measured. In order to reduce contact resistance, silver conductive paste was applied to the two measuring ends of the sample. The electrical conductivity σ [Siemens/meter] was calculated using the following Equation:

(1) $$\sigma =\frac{l}{\mathrm{RA}}\;\left[\frac{S}{m}\right]$$

where R is the electrical resistance of the specimen; l and A are the sample length and cross-section area, respectively.

### 3.4. Tensile Properties

Tensile tests were conducted at 23 °C on a universal testing machine (Instron Inc., 5500R, Norwood, MA, USA) in a tensile configuration according to standard DIN EN ISO 527-2. Dog-bone shape (ISO 572-2 type 1B) samples were used for the testing. The testing speed was chosen to be 2 mm/min with a 10 kN load cell, a precision sensor-arm extensometer was used to determine the specimen strain.

### 3.5. Fracture Tests

The plane strain fracture toughness (K_Ic_) of the composites was determined experimentally at 23 °C by using compact tension (CT) samples under tensile loading conditions according to the Norm ISO 13586 and at strain rate of 0.2 mm/min. The thickness B and the width W of specimens were chosen to be 4 mm and 30 mm, respectively. The samples were tested in a universal testing machine (Instron Inc., 5500R, Norwood, MA, USA). Before, testing a notch was machined and then sharpened by tapping a fresh razor blade into the material, so that a sharp crack was initiated with a length a_o_ (0.45·W ≤ a_o_ ≤ 0.55·W). The fracture toughness K_Ic_ was then calculated by Equation (2), where F is the maximum force observed in the load-displacement curve, and a_o_ is the initial crack length for calculating α = a_o_/W and f (a_o_/W ) as follows [24]

(2) $$K_{Ic}=\frac{F}{B\sqrt{W}}\cdot f\left(a_{o}/W\right)$$

(3) $$f\left(\frac{a_{o}}{W}\right)=f\left(\alpha \right)=\frac{\left(2+\alpha \right)}{{\left(1-\alpha \right)}^{3/2}}\cdot \left(0.866+4.64\alpha -13.32\alpha^{2}+14.72\alpha^{3}-5.60\alpha^{4}\right)$$

The critical stress intensity factor K_Ic_, the elastic modulus E_t_ and Poisson’s ratio ν (~0.35) [25] enable us in calculating the critical energy release rate G_Ic_

(4) $$G_{Ic}=\frac{K_{Ic}^{2}\left(1-\upsilon^{2}\right)}{E_{t}}$$

### 3.6. Microscopy Studies

The fractured surfaces of the CT tested specimens were studied with the help of a scanning electron microscope (Zeiss GeminiSEM 300, Carl Zeiss GmbH, Oberkochen, Germany). Prior to scanning, the fractured surface of the samples were sputtered with a thin layer of gold for 120 s using a sputtering device. Transmission electron microscopy (TEM) samples were sectioned using a diamond knife (Diatome, Biel-Bienne, Switzerland) on an ultracut (EM UCT, Leica Microsystems, Vienna, Austria). Ultrathin sections (70 nm) were picked up on copper grids with carbon film. Grids were examined with a Transmission Electron Microscope (Talos F200S G2-Thermofisher-Eindhoven) at 200kV, equipped with a 4K·4K camera One View (Gatan, Paris (France)).

## 4. Results

Epoxy cured systems have very low electrical conductivities in the order of 10^−14^ to 10^−17^ S/cm, however, different fillers have higher conductivities based on their conductive nature. For example, carbon black has conductivity of 10^2^ S/cm, graphite 10^5^ S/cm and pitch-based carbon fibers have 10^3^ S/cm [26]. Nanofillers have exceptionally high electrical conductivity and MWCNTs conductivity lies in the range of 10^4^–10^8^ Ω^−1^m^−1^ [27]. For reference epoxy system and the system with 0.05 wt. % of CNT the value to resistance is too high and it’s beyond the measuring limit of the equipment. For 0.075 wt. % and 0.1 wt. % the conductivity of composite is measured as 0.00026 S/m and 0.00163 S/m respectively. Table 2 shows the conductivity of all CNT modified systems.

**Figure 1:**
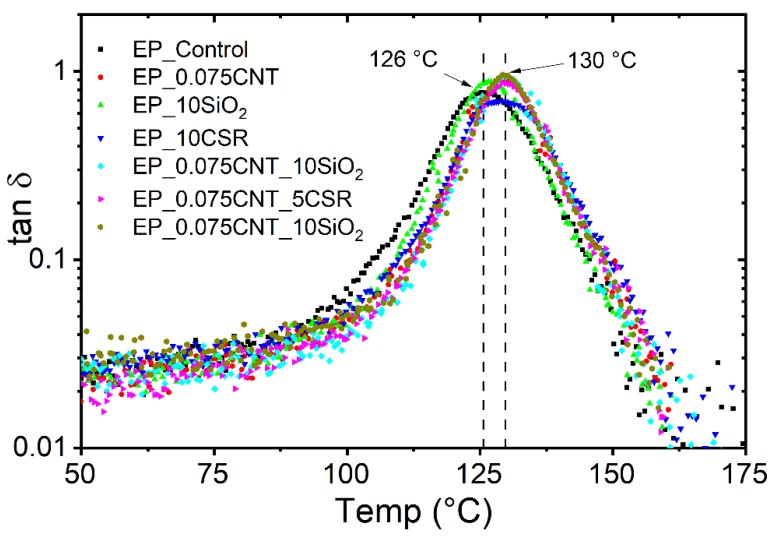
Dynamic mechanical analysis (DMA) (tanδ peak) curve to obtain glass transition temperature of reference and different modified epoxy system.

The T_g_ values of the epoxy reference system was measured as 120 °C. For all modified systems, the value of T_g_ lies in the range of 120 °C–125 °C, which shows that all the modifiers won’t affect the T_g_ of the obtained nanocomposites. Similarly, the T_g_ was also measured using the DMA (tanδ) and for all the systems values lies between 126 °C–130 °C. However, a difference was observed between the T_g_ values in both the techniques which may be due to the difference in the principle of measurements and heating rates used. Figure 1 shows the tanδ variation versus temperature for reference and modified systems. 

**Figure 2:**
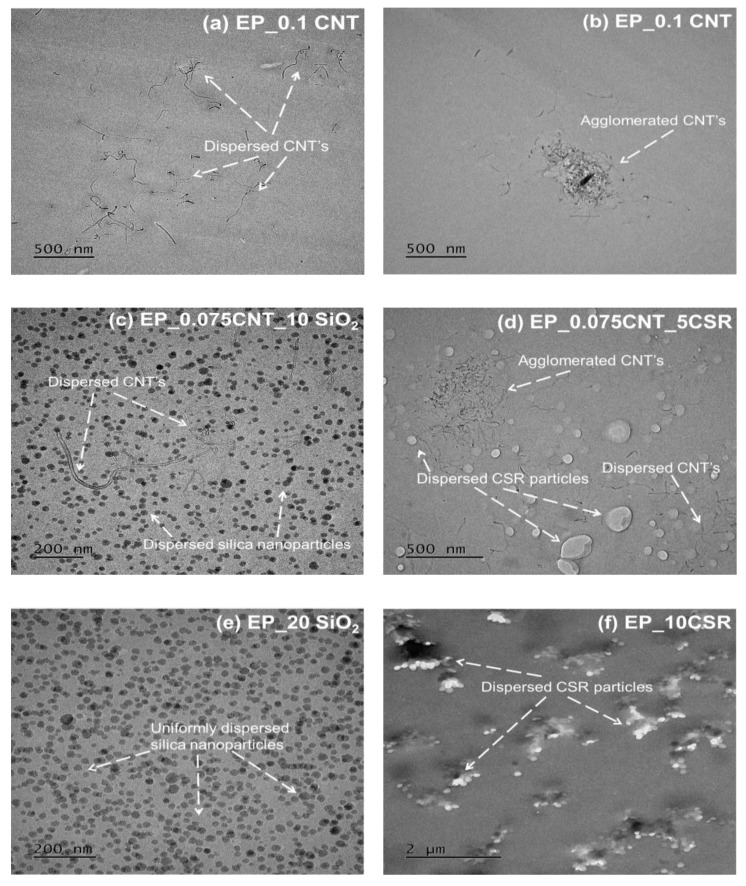
TEM images showing the dispersion of different fillers in modified epoxy systems. (**a**) EP_0.1 CNT; (**b**) EP_0.1 CNT; (**c**) EP_0.075 CNT_10 SiO_2_; (**d**) EP_0.075 CNT_5CSR; (**e**) EP_20 SiO_2_; (**f**) EP_10CSR.

The tensile properties of the reference and all modified systems are shown Table 3. A modulus of 2700 MPa and a tensile strength of 78 MPa were measured for the EP system. The addition of MWCNT’s reduces the tensile strength of the epoxies, which was expected due to possibility of agglomeration during the curing process while elastic modulus remains the same due to the low wt. % of CNT’s used. For the EP_20SiO_2_ system, the tensile strength and tensile modulus were measured as 90 MPa and 3200 MPa respectively which may be due to the uniform dispersion and effective adhesion between the epoxy matrix and SiO_2_ nanoparticles (see Figure 2c). The inclusion of the CSR particles decreases the modulus linearly with increasing filler wt. % and for EP_10CSR the tensile strength and tensile modulus were measured as 55 MPa and 2300 MPa respectively, which may be due to the soft core of the CSR particles. For hybrids, the three different systems were prepared with a concentration of CNT, SiO_2_ and CSR fixed at 0.075 wt. %, 10 wt. % and 5 wt. % respectively and excellent dispersion was observed in all the nanocomposites as seen in Figure 2c. Among hybrid systems the highest tensile strength of 82 MPa measured for EP_0.075CNT_10SiO_2_.

The K_Ic_, and G_Ic_ of the unmodified epoxy and epoxies modified with different nanofillers were measured in mode I using compact tension (CT) tests. The results are summarized in Table 3. The mean values for K_Ic_ and G_Ic_ of the unmodified epoxy were determined to be 0.55 MPa·m^1/2^ and 0.10 kJ/m^2^, respectively. For MWCNT’s modified epoxy systems maximum value of K_Ic_ (0.82 MPa·m^1/2^) and G_Ic_ (0.22 kJ/m^2^) was achieved at 0.1 wt. % of MWCNT. The maximum values of K_Ic_ = 1.72 MPa·m^1/2^ and the G_Ic_ = 1.13 kJ/m^2^ were measured for the EP_10CSR system revealing an increase by a factor of ~3 and 11.4 above the unmodified epoxy system. For SiO_2_ modified systems maximum values of K_Ic_ (1.60 MPa·m^1/2^) and G_Ic_ (0.70 kJ/m^2^) was achieved at 20 wt. %. Among hybrid systems maximum value of K_Ic_ = 1.75 MPa·m^1/2^ and the G_Ic_ = 1.00 kJ/m^2^ were measured for the EP_5CSR_10SiO_2_ system.

**Figure 3:**
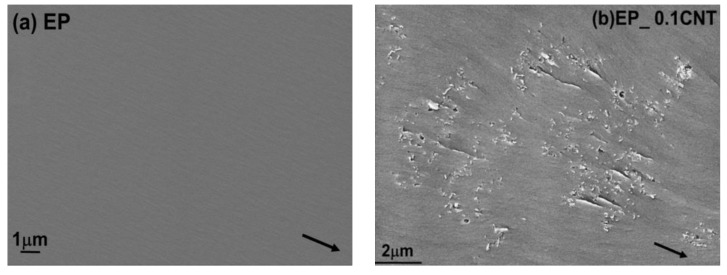

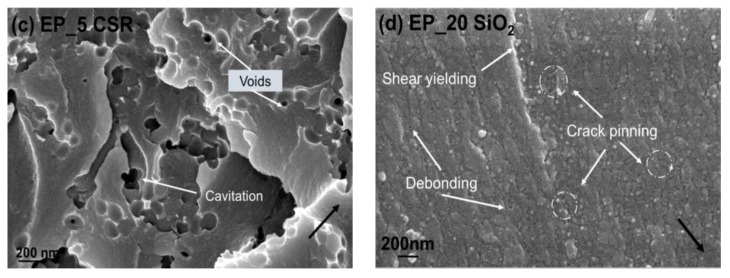
SEM micrographs of the fracture surface of the different reference and modified nanocomposite systems. Black arrow indicating the direction of crack propagation. (**a**) EP; (**b**) EP_0.1CNT; (**c**) EP_5CSR; (**d**) EP_20 SiO_2_.

**Figure 4:**
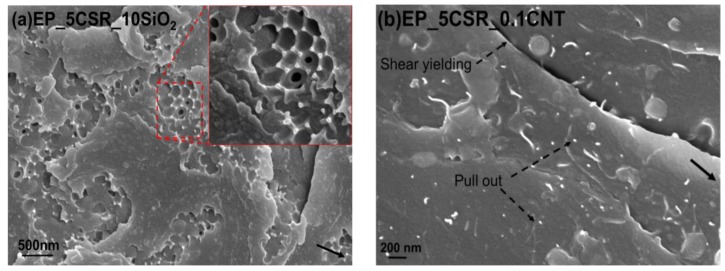
SEM micrographs of the fracture surface of the different hybrid systems. Black arrow indicating the direction of crack propagation. (**a**)EP_5CSR_10SiO_2_; (**b**) EP_5CSR_0.1CNT.

The toughening mechanisms responsible for the increase in fracture toughness due to the incorporation of different nano-fillers can be explained by analyzing the fracture surfaces of the unmodified and modified epoxy composites using SEM technique. The fracture surfaces for the unmodified epoxies, as shown in Figure 3a, for the amine-cured epoxy, show a smooth fracture for inherent brittle epoxy system, which indicates the brittle behaviour of epoxy system during the fracture process. For CNT modified nanocomposites a uniform dispersion was observed till 0.075 wt. %. At 0.1 wt. % traces of CNT agglomerates were found at the fracture surface as shown in Figure 3b. Debonded CNT’s were observed on the fracture surfaces. Therefore, debonding and fiber pull-out were considered to be the major toughening mechanisms linked with the CNT’s in epoxy hardener system. The Van der waals forces create an interactive attraction of the CNT’s they can drive CNT cluster into agglomerates in the epoxy matrix during curing process even after proper dispersion achieved during the mixing [28]. The presence of agglomeration was responsible for the reduction in the material’s toughness and its fracture energy [8]. This may be the sole reason, which was responsible for the CNT’s minor contribution on the toughening of brittle epoxy systems. Though the higher concentration of CNT’s increases the stiffness of the material but at the same time stress concentration caused by the CNT agglomerates initiated the crack and lead to fracture of the nanocomposites. The fractographic analysis of the fracture surface of SiO_2_ modified epoxy resin with the help of SEM and TEM can give an insight into the cause and location of failure, as well as the dispersion state of the particles within the epoxy matrix respectively. Figure 3d shows a close up of the crack surface in a nanocomposite containing 10 wt. % SiO_2_ nanoparticles, which reveals that fracture surface appears to be rougher as compared to unmodified system. Further, it shows the presence of voids around few silica nanoparticles based on these observations some nano and micro reinforcing mechanisms can be proposed. Some of these mechanisms include shear yielding, crack deflection, and particle crack pinning as specified by the small tails behind the particles. Similarly, for CSR modified systems river lines and ridges were observed on CSR modified systems along with voids which formed due to the cavitation of core of the CSR nanoparticles see Figure 3c. For hybrid systems, effective dispersion of nanoparticles were observed as seen in Figure 2c,d. The hybrid systems especially EP_5CSR_10SiO_2_ system shows rougher fracture surface due to their higher fracture energy as compared to other hybrid systems. Figure 4a reveals the presence of cavitation of CSR particles followed by void growth. It can be observed that silica nanoparticles were uniformly dispersed, and few cavities were observed around silica nanoparticles. The hybridization of silica and CSR interface provide synergy and results in increased fracture toughness. Heish et al [12] proposed that it may occur due to interaction between the particles of different Poisson’s ratio, elastic modulus which results in change of stress field in front of crack and provide enhanced degree of plastic deformation, which was previously established by many researchers that stress field interactions may enhance the intensity of plastic deformation mechanisms and extent of plastic zone [29,30]. Figure 4b shows the fracture surface of 5CSR_0.075CNT system showing the features like pull-out of CNT’s along with cavitation of CSR particles followed by plastic void growth.

**Figure 5:**
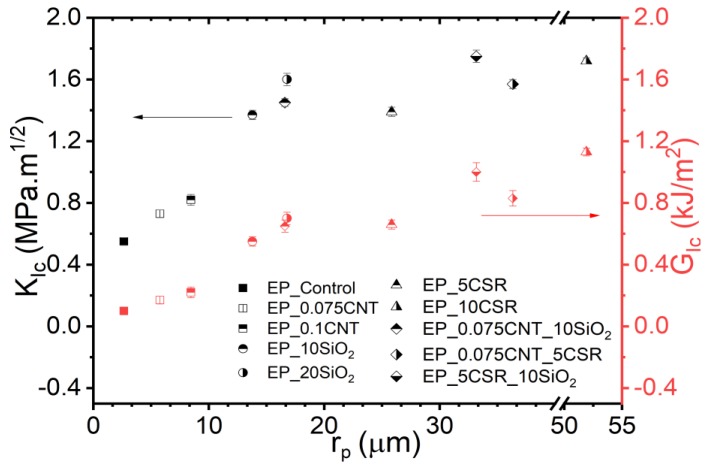
Fracture toughness and critical energy release rate of carbon nanotube (CNT), SiO_2_ and core-shell rubber (CSR)-modified epoxy systems as a function of plastic zone radius r_p_.

The plain strain dimension of plastic zone size can be quantified by Irwin’s model, assuming that the zone was circular and crack occurs in the matrix, by using Equation (5) [31] where K_Ic_ is the fracture toughness and $\sigma_{\mathrm{yt}}$ is the tensile true yield stress of the bulk polymer. A plastic zone radius of 2.64 µm was calculated for EP reference system. The maximum plastic zone size of 52 µm was calculated for EP_10CSR, and for all other modified systems the plastic zone size falls between these two limits (see Figure 5)

(5) $$r_{p}=\frac{1}{6\pi }{\left(\frac{K_{Ic}}{\sigma_{yt}}\right)}^{2}$$

The plastic zone was considerably larger than the radius of SiO_2_ and CSR nanoparticles see Figure 5. Therefore, these particles lie within the plastic zone and favor matrix toughening by events such as cavitation, plastic void growth, crack pinning, pull-out (for CNT’s) and crack deflection compelling the material to dissipate more energy before failure. Based on previous work [30,31] Equation (5) gave a good prediction of the size of the plastic deformation zone for the unmodified epoxies. However, for the filler modified epoxy systems, the degree of triaxiality of the stress around the crick tip is notably lower due to the formation of voids. This ultimately reduces the stress necessary for yielding of the epoxy matrix. Hence the size of plastic deformation zone of the particle modified epoxy systems is significantly larger than the plastic deformation size of unmodified systems [7,32,33]. 

**Figure 6:**
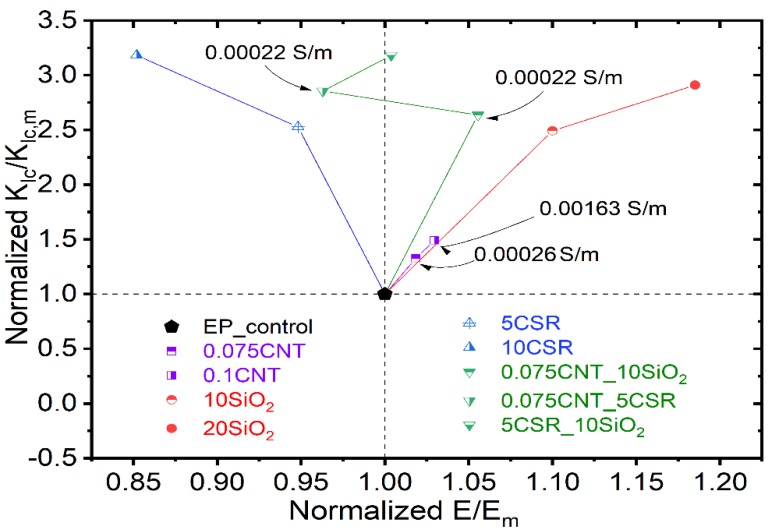
Normalized graph showing the relation between the normalized fracture toughness and normalized modulus of elasticity for different modified epoxy/amine systems in comparison to reference epoxy system. Electrically conductive systems are also mentioned in the figure.

It can be observed from the normalized graph (Figure 6) that, for CNT modified systems modulus and fracture toughness increases with increase in CNT concentration but the increase in values is very marginal. For SiO_2_ modified systems maximum increase of 3 and 1.2 times was observed for fracture toughness and tensile modulus respectively for EP_20SiO_2_ system. Similarly, for CSR modified systems an increase of 3.25 times was noted for EP_10CSR system. Hybrid systems, containing CNT have electrical conductivity ~0.0022 S/m with additional increase in fracture toughness values. Normalized map represents different properties for various nanocomposites making it convenient to select a typical system for certain property requirement.

## 5. Conclusions

The structure/property relationship of an amine hardener cured bisphenol A-based epoxy modified with CNT’s, rigid pre-dispersed SiO_2_ nano-particles, CSR nanoparticles, and a hybrid of CSR_CNT, SiO_2__CNT and CSR_SiO_2_ were investigated in terms of tensile, fracture mechanics properties, electrical properties, and thermo-mechanical properties, which further correlated with microstructural features and toughening mechanisms. The dispersion of all the fillers were uniform with few traces of agglomeration in case of CNT modified systems. The elastic modulus and ultimate tensile strength increased linearly with increasing wt. % of SiO_2_ nanoparticles due to the relative rigid nature of SiO_2_ nanoparticles and excellent adhesion between epoxy matrix and SiO_2_ nanoparticles. In the case of EP_CSR nanocomposites, the CSR nanoparticles were well dispersed with no traces of agglomeration. The elastic modulus and ultimate tensile strength were decreased with the addition of CSR nanoparticles, with increase in fracture toughness and fracture energy due to soft nature of rubber core in CSR. For CNT’s based epoxy nanocomposites, the electrical conductivity was threshold value was obtained for 0.075 wt. %. Further hybridization with CSR and SiO_2_ maintain the value of electrical conductivity with simultaneous improving the tensile and fracture properties. For EP_0.075CNT_10SiO_2_ system the increase of 163% was reported for fracture toughness with a minor enhancement of 5% in tensile strength and tensile modulus as compared to reference system. Of all the systems, the hybrids provide overall better performance in terms of tensile, fracture mechanics, electrical conductivity and thermo-mechanical properties as compared to those of single filler systems.

## Figures and Tables

**Table 1:** Nomenclature and composition of bulk epoxy-based composites. In notation (EP_x Y) EP denotes the reference epoxy/hardener system and x represents the wt. % used, and Y represents modifier, i.e. filler and dispersing DGEBA or DGEBF.

System	L1100 (gm)	Hardener 943 (gm)	Modifier (gm)
EP_Ref	142	33	-
EP_0.075CNT	116	28	5.65
EP_0.1 CNT	114	28	7.5
EP_10SiO_2_	87	26	38
EP_20SiO_2_	51.5	24	76.13
EP_5CSR	103	27	20.68
EP_10CSR	83.5	26	41.35
EP_0.075CNT_10 SiO_2_	81.78	26	5.51/37.73
EP_0.075 CNT_5 CSR	96.74	27	5.51/20.68
EP_5CSR_10SiO_2_	66.66	25	20.6/37.73

**Table 2:** Electrical conductivity of different modified epoxy nanocomposites.

Samples	Conductivity (S/m)
EP_Ref.	-
EP_0.05CNT	-
EP_0.075CNT	0.00026
EP_0.1CNT	0.00163
EP_0.075 CNT_10 SiO_2_	0.00022
EP_0.075 CNT_5 CSR	0.00021

**Table 3:** Tensile and fracture mechanics properties of reference and modified epoxy systems.

Systems	E_t_ [MPa]	σ_m_ [MPa]	ε_m_ [%]	K_Ic_ [MPa·m^1/2^]	G_Ic_ [kJ/m^2^]
EP	2700 (±15)	78 (±0.5)	6.8 (±0.1)	0.55 (±0.08)	0.10 (±0.07)
EP_0.075CNT	2750 (±35)	70 (±0.6)	6.2 (±0.2)	0.73 (±0.12)	0.17 (±0.06)
EP_0.1CNT	2730 (±45)	65 (±0.7)	5.9 (±0.1)	0.82 (±0.07)	0.22 (±0.05)
EP_10SiO_2_	2970 (±17)	85 (±0.9)	6.3 (±0.2)	1.37 (±0.08)	0.55 (±0.06)
EP_20SiO_2_	3200 (±10)	90 (±0.4)	6.1 (±0.3)	1.60 (±0.10)	0.70 (±0.07)
EP_5CSR	2560 (±12)	63 (±0.6)	5.2 (±0.4)	1.39 (±0.05)	0.66 (±0.03)
EP_10CSR	2300 (±24)	55 (±0.6)	4.7 (±0.2)	1.72 (±0.07)	1.13 (±0.04)
EP_0.075CNT_10SiO_2_	2850 (±25)	82 (±0.6)	6.1 (±0.3)	1.45 (±0.06)	0.65 (±0.04)
EP_0.075CNT_5CSR	2600 (±27)	60 (±0.6)	4.5 (±0.2)	1.57 (±0.04)	0.83 (±0.05)
EP_5CSR_10SiO_2_	2710 (±17)	70 (±0.8)	4.8 (±0.3)	1.75 (±0.08)	1.00 (±0.06)
